# Women and COVID-19: A One-Man Show?

**DOI:** 10.3389/fcvm.2020.596583

**Published:** 2020-12-16

**Authors:** Jef Van den Eynde, Karen De Vos, Kim R. Van Daalen, Wouter Oosterlinck

**Affiliations:** ^1^Department of Cardiovascular Diseases, University Hospitals Leuven, Leuven, Belgium; ^2^Faculty of Law, KU Leuven, Leuven, Belgium; ^3^Cardiovascular Epidemiology Unit, Department of Public Health and Primary Care, University of Cambridge, Cambridge, United Kingdom

**Keywords:** COVID-19, gender equality, human rights, public health, women

Coronavirus disease 2019 (COVID-19) severity and mortality have consistently been higher in men compared to women. The possible biological and behavioral factors underlying this difference have recently been analyzed by Capuano et al. (1). The ideas raised by the authors define a clear need for a more adequate approach to sex differences in case fatality rate. The higher mortality rate in men has indeed been described extensively in literature (2–4). However, the impact of the current pandemic reaches far beyond mortality rates. To tackle this pandemic effectively, an integrated response is essential (5). That is why in this article, we would like to draw attention to some of the main structural, psychological, social and economic impacts this pandemic has on women, as observed by academics, practitioners and international organizations.

Although we acknowledge gender to be complex, social, and non-binary, we will mainly focus on the impact of the current pandemic on women and refer to other publications about the impact on transgender and non-binary populations (6–8).

## The Current Lack of Sex-Disaggregated Data

**Figure 1:**
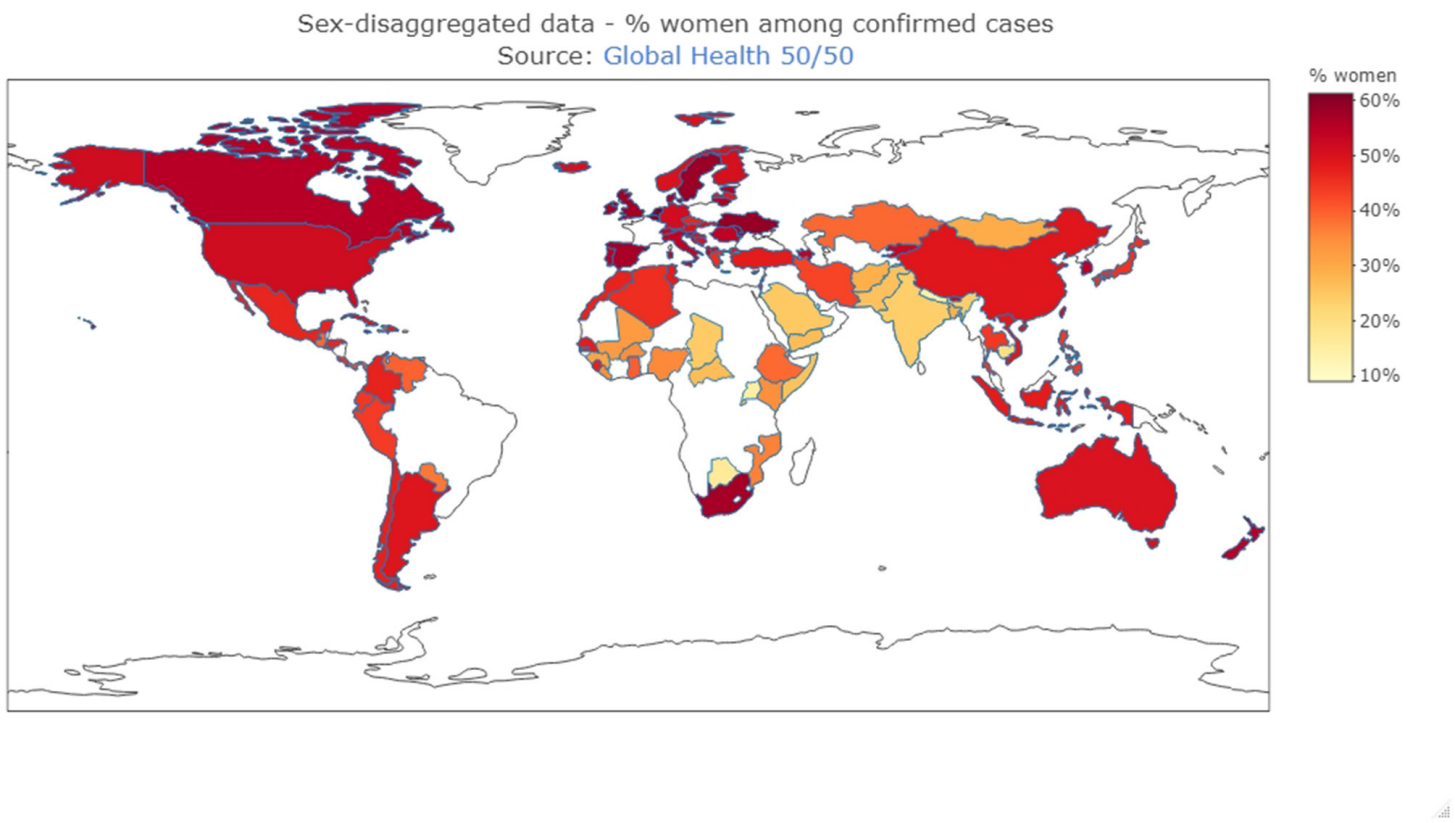
Sex-disaggregated data (Source: Global Health 50/50, https://globalhealth5050.org/covid19/sex-disaggregated-data-tracker/, 15/08/2020) Data are reported from the date that sex-disaggregated data was last available. The map was created using R Statistical Software (version 4.0.2. 2020-06-22, Foundation for Statistical Computing, Vienna, Austria), “plotly” package.

Sex- and gender-disaggregated data on COVID-19 confirmed cases are important in order to address gender disparities in COVID-19 health outcomes and ensure a gender-responsive approach. However, sex disaggregated data is lacking for most countries and gender disaggregated data is nearly absent. As of August 3, 2020, 18.07 million cases were reported worldwide. Data presented in Figure 1 (*n* = 8,587,718 sex-disaggregated cases), therefore, represent only 47.5% of all reported cases, highlighting the current lack of these valuable data. Furthermore, a striking difference in the percentage of women among confirmed cases is seen, with 60% in countries such as Belgium, the United Kingdom, and Canada, to 20% in countries such as the Central African Republic, Uganda, and India. Indeed, recent data show that among all persons tested for COVID-19 in the Central African Republic, only 26% were women.

## Infection Risk Among the Healthcare Workforce

Women face a higher risk of becoming infected during a pandemic because of their position in society as reported by the United Nations (UN) and the World Health Organization (WHO) (9–11). As doctors, nurses, midwives, and community health workers, women are overrepresented at the frontlines, making up 70% of the global health and social workforce (11). Particular issues are the global lack of personal appropriate protective equipment (PPE) and the fact that most PPE are based on a “*default man”* size providing a suboptimal barrier to most women and leaving them more exposed (12). Data from several outbreaks Ebola outbreaks and the SARS outbreak of 2003 demonstrate that nurses and other caretakers have been heavily infected in comparison to other groups in society (13).

## Social Impact

As a result of traditional social roles and stereotypes, women still act as the primary caregiver in households, globally spending three to four times more time on unpaid domestic work than men [The International Labor Organization (ILO)] (14). The additional care burden associated with childcare and homeschooling during lockdowns and the care for sick family members can lead to considerable health impacts including e.g., psychological stress. Usual coping mechanisms are limited, given the reduced contact with peers and the disruption of supportive networks. This especially hits single-parent households, of which the majority are headed by women (21% of households with children in the United States compared to 4% by men) (15). Furthermore, as a result, having less time for education, paid work, and career advancement, women can experience increased social inequality during this pandemic (15, 16). Stay-at-home measures together with financial and security concerns can put considerable strain on families, which in some situations can lead to domestic abuse and sexual violence. UN-reports show that violence against women and girls has increased by 25% in several countries and even doubled in some countries since the outbreak of COVID-19 (17).

## Economic Impact

Across the globe, women and girls earn less, have less access to educational opportunities, more often hold insecure jobs, and have limited access to financial resources and digital technology (18). Apart from deepening these existing inequalities, multiple studies show that the COVID-19 pandemic has a disproportionately large economic effect on women because the sectors in which they are most active are hard-hit (19). First of all, the manufacturing-and-retail industry has experienced large fallbacks in export and sales because of lockdown and distancing measures. The World Trade Organization (WTO) reports that female employees represent 80% of the workforce in ready-made garment production in Bangladesh, in which industry orders declined by 81% in April alone (20). Moreover, a larger share of women than men work in tourism and business travel which are highly disrupted by travel restrictions and will require a long recovery period (16, 18). Relying on face-to-face interactions, these occupations do not lend themselves to teleworking. Finally, this economic downturn will also be felt by female start-up entrepreneurs who are increasingly finding their way to micro, small and medium enterprises (MSMEs) (21). MSMEs tend to be the first businesses impacted in times of recession. Given the long-term economic impact that COVID-19 will have, protecting female entrepreneurship should be on the priority list of governments in order to build a faster and more inclusive growth during the economic recovery period.

## Human Rights

The Secretary General of the Council of Europe put it best: “While the virus is resulting in the tragic loss of life, we must nonetheless prevent it from destroying our way of life” (22). Human rights reflect the minimum standards necessary for people to live with dignity. While the COVID-19 crisis is fast becoming a socio-economic crisis it adds pressure on human rights. For women and girls, the problems identified form an undeniable increased threat to their right to life and right to health (23). Various international law instruments [e.g., The Universal Declaration of Human Rights, Art. 25 (24)] recognize the right to health as an inclusive right, encompassing a wide range of factors that help humans lead a healthy life (25). These factors include safe drinking water, safe food, sanitation, but also health-related education and information, the right to access to health care, and gender equality. As the UN state in their latest Policy Brief, the economic impact and prevalence of poverty among women, their experience of violence, their position in society, the limited power many women have over their sexual and reproductive lives, and their lack of influence in decision-making are social realities that adversely impact women's human rights and that should move to global action (9).

## A Way Forward

Prevention and response management is hindered when gendered impacts of outbreaks are ignored obscuring critical trends. In order to minimize these impacts, different steps should be undertaken. In Table 1 we provide a list of important recommendations made by international organizations.

**Table 1:** Recommendations for a more gender-sensitive approach to pandemics.

**Issue**	**Recommendation**
Lack of sex-disaggregated data	States, their partners and research institutions should collect, report, and analyze data on confirmed COVID-19 cases and deaths that are disaggregated by sex and age (10). The WHO provides global and national surveillance guidelines (10).
Higher risk of infection	Employers should be aware of the higher risk women face in the health and social domain and provide safe and decent working conditions. This can be monitored by workplace representatives, trade unions, and mutual control between employers (12).
Social impact	There should be more social awareness about the social impacts of pandemics. (In)formal protection and support services should be in place together with innovative solutions such as online fora and hotlines (16). Core health and education services and systems should be maintained (26).
Economic impact	Apart from tackling existing economic inequalities, (financial) support measures for businesses should be provided to prevent an economic downfall (16). Moreover, the value of women's unpaid care work should be recognized by including it in the formal labor market and redistributing unpaid family care equally.
Human rights	Decision-makers should be aware that outbreaks affect groups differently and ensure a gender-responsive intersectional response to the COVID-19 pandemic (that recognizes the realities of different genders and addresses these) in policies, program development, implementation etc. Increased participation of women in decision-making will help establish adaptive responses to these realities (27). Inclusivity and diversity in decision-making should be ensured reflecting the population they represent. Existing women's and youth rights networks should be engaged to support connectivity and vital information flow (26).

## Conclusion

Gendered differences of COVID-19 are present not only at the biological level, but also at the psychological, social and societal level. Although literature shows that men are clearly predisposed to COVID-19 related mortality, women are just as well victimized, albeit in a different way. The current pandemic painfully highlights that gender inequality is still insufficiently addressed in our society. Public health should never be a predominantly men affair mainly focusing on the male body—a one-man show. In contrast, more gender-sensitive approaches that take into account different physical, mental, and social needs across the full gender spectrum are indispensable to guarantee optimal well-being of all.

## Author Contributions

JV and KD conceived and wrote the manuscript. KV and WO critically revised the manuscript and provided important intellectual contribution. All authors contributed to the article and approved the submitted version.

## Conflict of Interest

The authors declare that the research was conducted in the absence of any commercial or financial relationships that could be construed as a potential conflict of interest.
